# Keratinocyte HIF-1α Orchestrates Imiquimod-Induced Psoriasiform Inflammation by Promoting Type 3 Inflammation

**DOI:** 10.3390/biomedicines14010065

**Published:** 2025-12-28

**Authors:** Dohyeon Ku, Kwonik Oh

**Affiliations:** 1 Department of Pathology, Hallym University College of Medicine, Chuncheon 24252, Republic of Korea; dhku@hallym.ac.kr; 2 Institute of Medical Science, Hallym University College of Medicine, Chuncheon 24252, Republic of Korea

**Keywords:** Psoriasis, imiquimod, hypoxia-inducible factor, IL-17

## Abstract

Psoriasis is a chronic inflammatory skin disease driven by the IL-23/IL-17 axis and characterized by keratinocyte hyperproliferation, epidermal thickening, and immune infiltration. While immune cell-intrinsic roles of hypoxia-inducible factor-1α (HIF-1α) have been reported, the contribution of keratinocyte HIF-1α remains less clear. In this study, we investigated epithelial HIF function in murine models of skin inflammation using keratinocyte-specific HIF-1α knockout (K14-Cre Hif1a^fl/fl^) mice. HIF-1α deficiency attenuated epidermal hyperplasia and type 3 inflammation in the imiquimod (IMQ)-induced psoriasiform model but had little effect in DNFB-induce contact hypersensitivity and MC903-induced atopic dermatitis model. Flow cytometry of draining lymph nodes revealed reduced frequencies of inflammatory cells including IL-17-producing γδ T cells in HIF-1α-deficient mice. In IMQ-treated skin, HIF-1α deficiency led to reduced *Il17*, *Il23* and neutrophil-attracting chemokine transcript levels and diminished Ly6G^+^ neutrophil infiltration. These findings identify keratinocyte HIF-1α as a central regulator of psoriasiform inflammation and suggest that epithelial HIF signaling could be a potential therapeutic target for psoriasis.

## 1. Introduction

Psoriasis is a chronic, immune-mediated inflammatory skin disease affecting approximately 2% of population in Europe and North America [1,2]. Clinically, psoriasis presents as erythematous, scaly plaques, histologically marked by epidermal hyperplasia, parakeratosis, and dense infiltration of immune cells within the dermis and epidermis [3]. Over the past two decades, substantial progress has been made in elucidating the immunopathogenesis of psoriasis, identifying the IL-23/IL-17 cytokine axis as a central driver of disease [4,5,6]. Therapeutic blockade of IL-23 or IL-17 leads to profound clinical improvement, underscoring the pivotal role of this pathway in human disease [7,8].

Psoriasis arises from complex interactions between the immune system and epidermal keratinocytes. While T helper 17 (Th17) and γδ T cells are recognized as key sources of IL-17 [9,10], keratinocytes are not passive structural cells but active participants that shape cutaneous inflammation. They secrete cytokines (IL-1, IL-36, TNF), antimicrobial peptides (S100 proteins, LL-37), and chemokines that recruit neutrophils and T cells, thereby amplifying inflammation [11,12]. Consequently, dissecting keratinocyte-intrinsic signaling pathways is critical to fully understanding psoriasis pathogenesis.

HIFs are transcription factors that orchestrate cellular adaptation to low oxygen tension. The HIF-1α subunit, stabilized under hypoxic or inflammatory conditions, translocates to the nucleus to induce genes involved in glycolysis, angiogenesis (e.g., VEGF), and inflammation [13,14]. Importantly, HIF-1α stabilization is not restricted to classical hypoxia; inflammatory cytokines, reactive oxygen species (ROS), and microbial products can also activate it, linking HIF signaling to immunometabolic reprogramming [15,16,17]. In immune cells, HIF-1α has well-established roles. It promotes Th17 differentiation while suppressing regulatory T cell (Treg) development through direct transcriptional regulation of *Rorc* and *Il17* [18]. In innate immune cells, HIF-1α supports glycolytic metabolism required for effector functions of macrophages and neutrophils [19]. However, the role of HIF-1α within keratinocytes remains poorly understood. Keratinocytes reside in a relatively hypoxic environment, particularly during inflammation when oxygen consumption increases due to infiltrating immune cells [20,21]. Under such conditions, HIF-1α stabilization in keratinocytes may act as an amplifier of inflammatory signaling. Yet whether keratinocyte HIF-1α contributes to the initiation or propagation of cutaneous inflammation remains unclear.

In this study, we employed keratinocyte-specific HIF-1α knockout (K14-Cre; Hif1a^fl/fl^) mice to define the role of epithelial HIF-1α in three distinct murine models of skin inflammation: (1) 2,4-dinitrofluorobenzene (DNFB)-induced contact hypersensitivity, (2) MC903-induced atopic dermatitis-like inflammation (type 2 inflammation), and (3) imiquimod (IMQ)-induced psoriasiform dermatitis (type 3 inflammation). We found that keratinocyte HIF-1α was dispensable for DNFB- and MC903-induced responses but essential for IMQ-induced psoriasiform inflammation. In the IMQ model, loss of keratinocyte HIF-1α markedly reduced erythema and epidermal thickening, suppressed activation of the IL-23/IL-17 axis, decreased IL-17-producing T cells, and attenuated neutrophil infiltration. These findings identify keratinocyte HIF-1α as a critical regulator of psoriatic inflammation.

## 2. Materials and Methods

### 2.1. Mice

Floxed Hif-1α (B6.129-Hif1atm3Rsjo/J) and K14Cre (B6N.Cg-Tg (KRT14-cre) 1Amc/J) mice were obtained from The Jackson Laboratory (Bar Harbor, ME, USA). Keratinocyte-specific HIF-1α knockout mice (K14Cre Hif1a^fl/fl^) were generated by crossing K14Cre transgenic mice with floxed Hif-1α mice (Jackson Laboratory). Wild-type littermates served as controls. Mice were housed under SPF conditions. All experiments were approved by the Institutional Animal Care and Use Committee.

### 2.2. Induction of Skin Inflammation

DNFB model: Mice were sensitized with 0.5% DNFB (Sigma-Aldrich, St. Louis, MO, USA) on abdominal skin and challenged with 0.2% DNFB on ears. Ear thickness was measured on day 8. We performed the induction of contact hypersensitivity in mice as described previously [22].

MC903-induced murine AD model: MC903 (calcipotriol, Sigma-Aldrich) was dissolved in EtOH and topically applied on one or both ears. Each ear was sensitized daily with 0.5 nmol of MC903 or the same volume of EtOH (control) for 14 days unless specified otherwise. During the period of MC903 treatment, ear thickness was measured using a micrometer (Mitutoyo, Kanagawa, Japan).

IMQ model: 62.5 mg of 5% IMQ cream (Aldara) was applied daily to shaved back skin for 4 days. Erythema was assessed by two independent investigators using a 0–4 scoring system, in which 0 indicates none, 1 slight, 2 moderate, 3 marked, and 4 very marked erythema [23]. Dorsal skin and draining lymph nodes were harvested after 4-day treatment for further study.

### 2.3. Histology

Skin tissues were fixed with 10% formalin and embedded in paraffin. Tissues were sectioned into 5-μm sections and stained with hematoxylin and eosin to evaluate histopathological changes. Epidermal thickness was quantified in more than five fields per slide using Image J, with four to five slides analyzed per mouse.

### 2.4. Tissue Preparation and Flow Cytometry

Single-cell suspensions were prepared from lymph nodes and skin. Skin tissues were minced and digested in 2 mL HBSS containing 0.1 mg/mL DNase I and 0.1 mg/mL Liberase TL (Sigma-Aldrich) for 1 h at 37 °C. The suspension was then passed through a cell strainer (SPL, Seoul, Korea). For surface staining, the cells were stained with antibodies for 30 min at 4 °C in the dark. For intracellular staining, the cells were stained using the Foxp3 Staining Buffer Set (Thermo Fisher Scientific, Waltham, MA, USA). For cytokine analysis, we cultured cells for 4 h in the presence of PMA/ionomycin plus monensin (BD Biosciences, San Jose, CA, USA) before intracellular cytokine staining unless otherwise specified. Data were acquired using FACS Canto-II (BD Biosciences) and analyzed using FlowJo software (version 10, BD Biosciences).

### 2.5. Quantitative RT-PCR (RT-qPCR)

We isolated RNA using the RNeasy Mini kit (Qiagen, Germantown, MD, USA) or TRIzol (Thermo Fisher Scientific Korea, Seoul, Korea), and reverse-transcribed it into cDNA using QuantiTect Reverse Transcription kit (Qiagen). We normalized all data to actin. We checked non-specific amplification by the use of melting curves and agarose gel electrophoresis. The sequences of primers (Genotech, Daejon, Korea) were as follows.

Il17a forward, 5′-ACTACCTCAACCGTTCCACGTC-3′;

Il17a reverse, 5′-ATGTGGTGGTCCAGCTTTCC-3′;

Ifng forward, 5′-GATGCATTCATGAGTATT GCCAAGT-3′;

Ifng reverse, 5′-GTGGACCACTCGGATGAGCTC-3′;

Cxcl1 forward, 5′-TGAGCTGCGCTGTCAGTGCCT-3′;

Cxcl1 reverse, 5′-AGAAGCCAGCGTTCACCAGA-3′;

Cxcl2 forward, 5′-GAGCTTGAGTGTGACGCCCCCAGG-3′;

Cxcl2 reverse, 5′-GTTAGCCTTGCCTTTGTTCAGTATC-3′;

actin forward, 5′-CATCCGTAAAGACCTCTATGCCAAC-3′;

actin reverse, 5′-ATGGAGCCACCGATCCACA-3′.

### 2.6. Statistics

A two-tailed, unpaired, Student *t*-test was used to calculate the statistical significance of differences between groups. The P values are represented as follows: ***, *p* < 0.001; **, *p* < 0.01; *, *p* < 0.05, whereas NS, not significant, is used to denote *p* values > 0.05. Error bars indicate s.d.

## 3. Results

### 3.1. Keratinocyte HIF-1α Is Essential for Psoriasiform Inflammation

**Figure 1:**
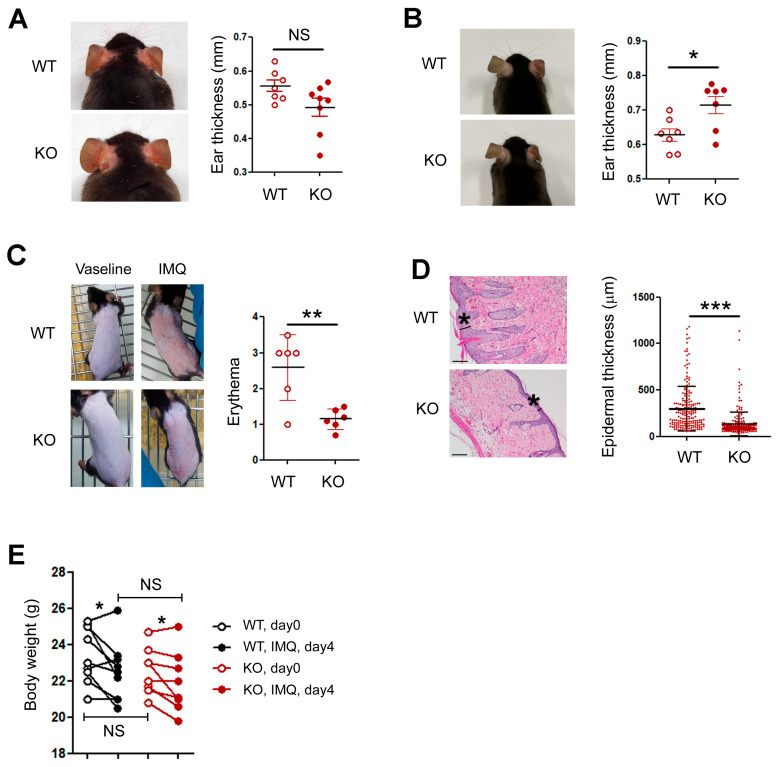
Keratinocyte HIF-1α is essential for psoriasiform inflammation. (**A**) Representative images of ears treated with DNFB (left). Ear thickness measured on day 8 showed no significant difference in DNFB-induced dermatitis between wild-type (WT) and keratinocyte-specific HIF-1α knockout (KO) mice (right). (**B**) MC903-induced ear swelling was increased in KO mice compared with WT mice. MC903 was applied to the right ear only. (**C**) IMQ-induced erythema was significantly reduced in KO mice compared with WT mice. Vaseline was used as a control. (**D**) Representative hematoxylin- and eosin-stained sections and quantitative analysis of epidermal thickness (indicated by asterisks) show reduced epidermal hyperplasia in KO mice. Epidermal thickness was measured in more than five fields per slide using Image J, with four to five slides analyzed per mouse. Pooled data collected from WT and KO mice (*n* = 6, each) are shown on the right, with each dot representing an individual measurement. (**E**) Changes in body weight before and after IMQ treatment in WT and KO mice. Pooled data are shown in A, B, C (right panel), and E, with each circle corresponding to an individual mouse. Data are presented as the mean ± SD. NS, not significant; ***, *p* < 0.001; **, *p* < 0.01; *, *p* < 0.05.

To delineate the role of keratinocyte-derived HIF-1α in cutaneous inflammation, we employed three murine models representing distinct immune responses: DNFB-induced contact dermatitis, MC903-induced atopic dermatitis-like inflammation, and IMQ-induced psoriasiform inflammation. Each model was compared between wild-type (WT) and keratinocyte-specific HIF-1α knockout (KO) mice. In the DNFB-induced model, ear appearance and swelling were comparable between WT and KO mice (Figure 1A). In the MC903 model, KO mice exhibited a modest but statistically significant increase in ear thickness (Figure 1B). In contrast, in the IMQ-induced psoriasiform model, KO mice displayed markedly reduced erythema (Figure 1C) and significantly attenuated epidermal hyperplasia (Figure 1D). Both WT and KO mice lost weight to a similar extent (Figure 1E). Collectively, these results indicate that keratinocyte HIF-1α differentially regulates inflammatory responses depending on the immune context and is indispensable for IMQ-driven psoriasiform inflammation. Based on these findings, we subsequently focused on the IMQ model to investigate the role of keratinocyte HIF-1α.

### 3.2. Suppression of IL-23/IL-17 Axis and Neutrophil Recruitment in HIF-1α KO

**Figure 2:**
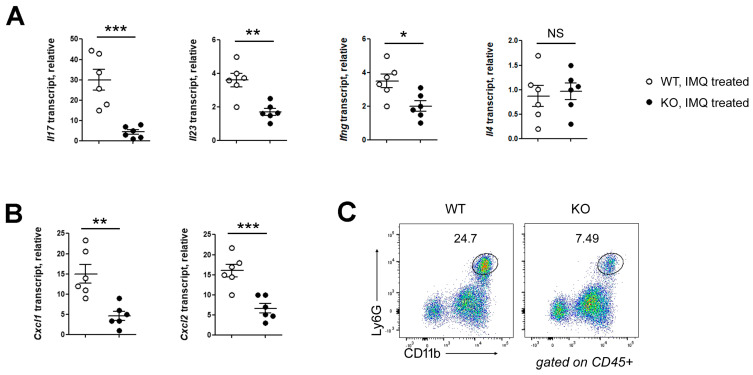
Keratinocyte HIF-1α drives IL-23/IL-17 cytokine production and neutrophil recruitment. (**A**) Quantitative PCR analysis of *Il17a*, *Il23a*, *Ifng*, and *Il4* expression in inflamed skin tissues. (**B**) *Cxcl1* and *Cxcl2* transcripts were reduced in KO skin. (**C**) Flow cytometric analysis of skin treated with IMQ. The percentages of the neutrophil subset (CD11b^+^Ly6G^+^) in WT and KO are shown. Pooled data are shown in A and B, with each circle representing an individual mouse. Data are presented as the mean ± SD. NS, not significant; ***, *p* < 0.001; **, *p* < 0.01; *, *p* < 0.05.

To elucidate the downstream inflammatory pathways regulated by keratinocyte HIF-1α, we examined the expression of pro-inflammatory cytokines in lesional skin. Both type 3 (IL-17 and IL-23) and type 1 (IFN-γ) cytokines were significantly downregulated in HIF-1α KO mice compared with WT controls (Figure 2A). Moreover, the expression of neutrophil-attracting chemokines Cxcl1 and Cxcl2 was markedly reduced in KO skin (Figure 2B). Flow cytometric analysis further confirmed a substantial decrease in Ly6G^+^CD11b^+^ neutrophil infiltration in KO mice relative to WT counterparts (Figure 2C). Together, these findings demonstrate that keratinocyte HIF-1α promotes psoriasiform inflammation by amplifying the IL-23/IL-17 axis and enhancing neutrophil infiltration.

### 3.3. Keratinocyte HIF-1α Deficiency Reduces IL-17-Producing T Cells

**Figure 3:**
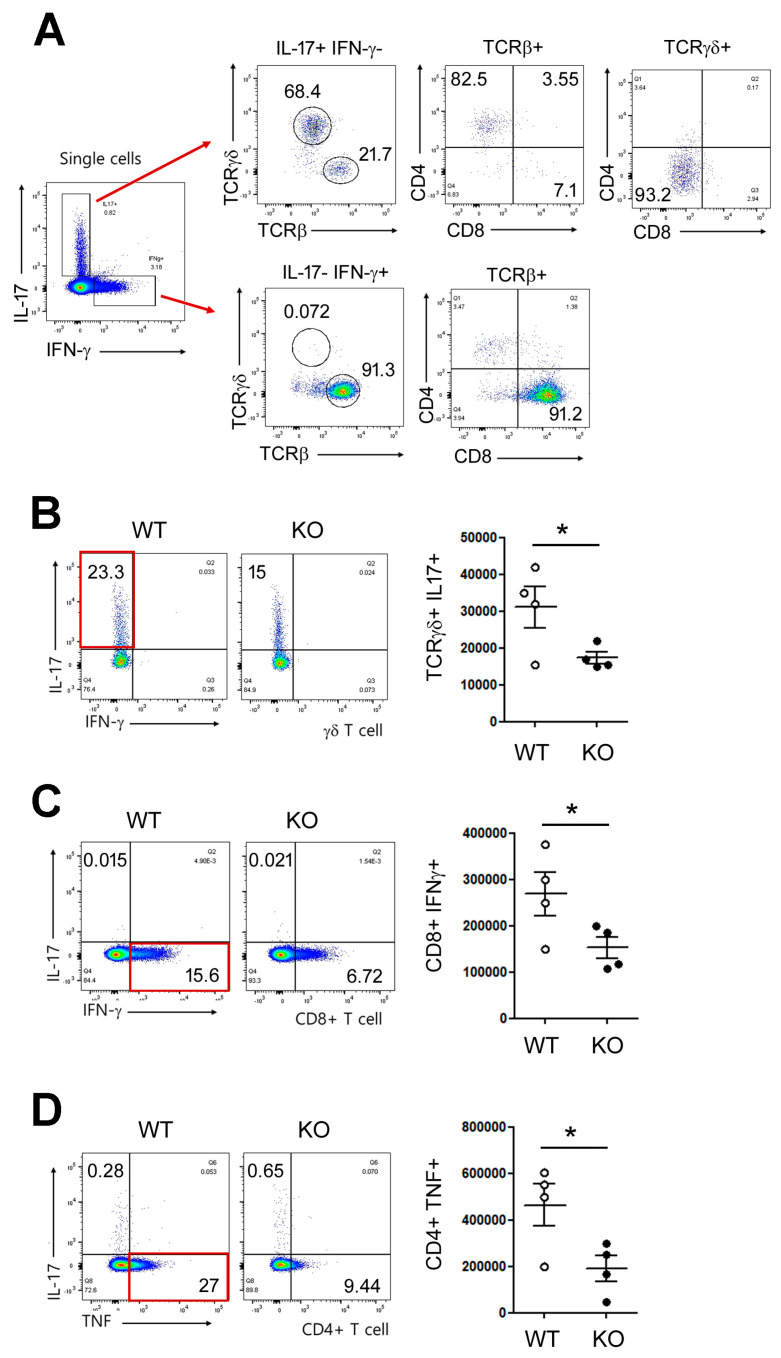
Keratinocyte HIF-1α deficiency reduces IL-17-producing T cells after IMQ treatment. (**A**) Gating strategy used to identify IL-17- and IFN-γ- producing cell populations. (**B**–**D**) Frequencies (left) and absolute numbers (right panel) of IL-17-producing γδ T cells (**B**), IFN-γ-producing CD8^+^ T cells (**C**) and TNF-producing CD4^+^ T cells (**D**) in WT and KO mice after IMQ treatment. Pooled data from B, C and D are shown, with each circle representing an individual mouse. Data are presented as the mean ± SD. *, *p* < 0.05.

Next, we performed flow cytometric analysis of draining lymph nodes after IMQ treatment, which revealed that IL-17 and IFN-γ production was strongly induced in γδ T and CD8^+^ T cells, respectively (Figure 3A). In contrast, HIF-1α KO mice displayed a significant reduction in the frequency and number of TCRγδ^+^IL-17^+^ T cells (Figure 3B), CD8^+^IFN-γ^+^ T cells (Figure 3C) and CD4^+^TNF^+^ T cells (Figure 3D). These results indicate that keratinocyte HIF-1α indirectly supports the expansion or maintenance of type 1 and 3 cytokine-producing T cells during psoriatic inflammation.

### 3.4. HIF Inhibitor Treatment Ameliorates IMQ-Induced Skin Inflammation

**Figure 4:**
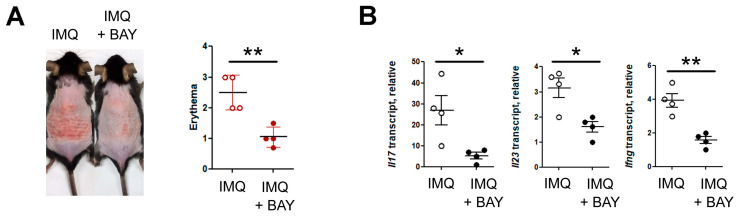
Pharmacologic inhibition of HIF alleviates psoriasiform inflammation. (**A**) Representative images showing reduced erythema following topical BAY 87-2243 application (IMQ + BAY). (**B**) Quantitative RT-PCR analysis of inflammatory cytokines (*Il17a*, *Il23a*, *Ifng*) in skin treated with IMQ or IMQ + BAY. Pooled data are shown, with each circle representing an individual mouse. Data are presented as the mean ± SD. **, *p* < 0.01; *, *p* < 0.05.

Our findings suggest that keratinocyte HIF signaling contributes to IMQ-induced inflammation, which led us to test the effect of topical application of a HIF inhibitor (BAY 87-2243, [24]). Topical administration of BAY 87-2243 markedly reduced erythema compared with vehicle-treated controls (Figure 4A). Consistent with these observations, quantitative RT–PCR revealed substantial downregulation of IL-17, IL-23 and IFN-γ transcripts in BAY-treated skin (Figure 4B), indicating that pharmacologic inhibition of HIF signaling recapitulates the phenotype observed in keratinocyte-specific HIF-1α knockout mice.

## 4. Discussion

Our results identify keratinocyte HIF-1α as a crucial amplifier of psoriasiform inflammation and a potential therapeutic target for psoriasis. Using keratinocyte-specific HIF-1α knockout mice and pharmacologic inhibition of HIF, we demonstrate that epithelial HIF-1α promotes IL-23/IL-17-driven inflammation and epidermal hyperplasia in the IMQ model. These findings reveal that keratinocyte HIF signaling functions not merely as a metabolic sensor but as an active regulator of inflammatory crosstalk between epithelial and immune compartments.

Psoriasis is fundamentally an IL-23/IL-17-driven disease, wherein keratinocytes and immune cells form a self-amplifying loop. IL-17 produced by γδ T and Th17 cells stimulates keratinocytes to release antimicrobial peptides, cytokines, and chemokines such as CXCL1 and CXCL8, thereby attracting neutrophils and sustaining inflammation [9,10,11]. Our findings imply that loss of keratinocyte HIF-1α disrupts this circuit: IL-17^+^ γδ T cells were reduced, as were *Il23* and *Cxcl1*/*Cxcl2* transcripts. These findings suggest that keratinocyte HIF-1α acts as an upstream regulator that converts local stress (e.g., TLR7 stimulation by IMQ [25] or inflammatory hypoxia) into proinflammatory cytokine production, reinforcing the IL-23/IL-17 axis.

Several studies have demonstrated that HIF signaling is closely associated with the expression of neutrophil-attracting CXC chemokines, including CXCL1 and CXCL2, in various tissues and pathological conditions, such as atherosclerosis [26], colitis-associated cancer [27], acute pancreatitis [28], and pulmonary infection [29]. Since several putative HREs have been identified in the CXCL1 promoter [27,29], HIF appears to regulate CXCL1 expression through both direct transcriptional mechanisms and indirect pathways. Given that IMQ-induced psoriasiform inflammation relies on IL-23/IL-17-driven type 3 immunity and neutrophil infiltration, it is plausible that keratinocyte HIF-1α contributes to the inflammatory milieu at least in part by modulating the expression of CXCL1/2. Thus, the reduced inflammatory phenotype observed in keratinocyte-specific HIF-1α-deficient mice may reflect impaired chemokine-mediated recruitment of neutrophils in the IMQ model.

Recent studies have demonstrated the relationship between IL-17 and HIF-1α in injured skin [30] and psoriasis, showing that the IL-17/HIF-1a signaling axis can drive a glycolysis program in epithelial cells and thereby sustain skin inflammation [31]. Consistent with these reports, our study examined the role of keratinocyte HIF-1α across various skin inflammation models and revealed broader regulatory effects in HIF-1α KO mice. One of the striking findings was that keratinocyte HIF-1α appeared to exert opposing functions depending on the inflammatory context—suppressing type 2 inflammation while promoting type 3 inflammation. Given that glycolytic metabolism is crucial for both inflammations, these results suggest that HIF-1α may regulate skin inflammation through additional, glycolysis-independent mechanisms.

In summary, our results establish keratinocyte HIF-1α as a key epithelial amplifier of psoriatic inflammation that links metabolic reprogramming with IL-23/IL-17-mediated immune activation. Pharmacologic inhibition of HIF signaling alleviates IMQ-induced dermatitis, supporting the concept that epithelial HIF-1α represents a viable and selective therapeutic target for psoriasis.

## Data Availability

The datasets used in the study are available from the corresponding author upon request.
